# Rehabilitation: The Missing ‘Medicine’ for Recovering From Zoonotic Illnesses?

**DOI:** 10.7759/cureus.81202

**Published:** 2025-03-25

**Authors:** Musa Kiyani, Rebecca Fam Qi Hui

**Affiliations:** 1 General Medicine, Sengkang General Hospital, Singapore, SGP; 2 Physiotherapy, Sengkang General Hospital, Singapore, SGP

**Keywords:** covid-19, neuroinflammation, physiotherapy, rehabilitation, zoonoses

## Abstract

Outbreaks of zoonotic illnesses, from previously neglected monkeypox to the novel pandemic of coronavirus disease 2019 (COVID-19), herald a new contagious manifestation of diseases that threaten global health safety, dwindle scarce resources, and generate hurtful stigma. Clinical and public health interventions, such as isolation and quarantine of the sick and their contacts, induction of vaccine-derived immunity, and the use of therapeutics, often overlook long-term musculoskeletal and neurological complications. Here, we employ a biopsychosocial lens to elucidate the theoretical underpinnings and broader practical aspects of rehabilitation in our experience of caring for COVID-19 and other patients in Singapore’s public hospitals. We discuss the often-overlooked complications of these illnesses, including sarcopenia and neuroinflammation. These can delay rehabilitation in the most vulnerable and bring to the forefront the drawbacks of policies that restrict mobility and socialization.

## Editorial

‘Neuroinflammation’, hyper-coagulability, and other inflammatory states often precede delirium, while prolonged stay in the hospital, opioid and anticholinergic use, and other iatrogenic etiologies predispose recovering patients to cognitive dysfunction and functional decline [1]. Advanced age and harboring comorbidities can further elevate disease severity and mortality risk in coronavirus disease 2019 (COVID-19) [2]. In our practice, it is often the geriatric population with a prolonged course of disease or social factors that prevent their early discharge who are exposed to the full gamut of adverse physical and mental outcomes. We recall an elderly patient with multiple chronic illnesses who was admitted to our hospital for worsening dyspnea, eventually attributed to both critical COVID-19 infection and pulmonary tuberculosis. He was supported on a mechanical ventilator for a month in the Intensive Care Unit before transitioning to care in the general ward. Symptoms of exercise intolerance and fatigue affected his participation during therapy, resulting in him gradually developing severe, extensive muscle atrophy. On verticalization, his neuro-vestibular and cardiovascular systems were also challenged as symptoms of dizziness and breathlessness would set in rather quickly. This patient displayed barriers to mobilization in the form of platypnea-orthodeoxia syndrome, with positional dyspnea in an upright position. Keeping this in mind, our physiotherapists introduced graded verticalization - supine MOTOmed followed by the tilt table, slow increase in the duration of sitting out of bed, and eventual ambulation. Anhedonia, likely exacerbated by the prolonged hospitalization and detachment from his family, continued to adversely affect participation during therapy sessions. While our experience has shown that most recovering patients benefit from extensive post-acute care rehabilitation, occasionally, as in this case, the initial resistance in reversal of respiratory and other symptoms can parallel subsequent care needs and rehabilitation potential. 

Nationally, Singapore’s strategy of close surveillance and containment to limit the spread of COVID-19 has come at the expense of early and extensive rehabilitation. Previously, group activities involving the elderly in senior group homes, day care, and day rehabilitation centers were forced to cease, while outpatient rehabilitation services were heavily curtailed. For those not sick enough to be admitted to an acute hospital, community facilities were arranged while others recovered at home. As a result, we have seen how recovering COVID-19 patients and their caregivers struggle to cope with changes in their daily routines, which presumably exacerbate sedentary behaviors at home. There are many examples of our community ambulant patients who would become more homebound post-hospitalization, resulting in decreased physical activity and caloric insufficiency. A vicious cycle would ensue since inactivity and poor nutrition worsen sarcopenia, frailty, and fall risk, all of which are known contributors to disability amongst the elderly [3]. 

The road to achieving herd immunity in Singapore was wrought with vaccine hesitancy due to concerns about safety and adverse reactions. An elderly, unvaccinated woman under our care was admitted due to immobility after recurrent falls. Mandatory diagnostic testing for COVID-19 at the time of admission meant that this lady was not only infected with the disease, much to her and her family’s dismay, but now needed to be isolated, monitored in an acute hospital for a few days, and then eventually transferred to a COVID-19 treatment facility (CTF). Our colleagues, mostly locum physiotherapists, were tasked to review her occasionally at the CTF, until she was brought back to our acute hospital due to inability to reach her premorbid function within a week and difficulties in settling discharge logistics. Arranging caregiver training proved to be a challenge as her caregiver was unvaccinated, further prolonging her stay in the inpatient setting. Although she was eventually discharged after almost a month of extensive rehabilitation, small case studies in Singapore have highlighted significant delayed health consequences for those without good caregiving support in the community. For instance, a case report from the National University Hospital in Singapore follows an 84-year-old retired schoolteacher who was diagnosed with ‘worsening depression’ and sent to a Geriatrics clinic [4]. He was later found to have a prolonged variant of delirium with rapid cognitive and functional decline, likely exacerbated by lockdown restrictions and delay in seeking medical care. A case series also demonstrated how geriatric patients with COVID-19 and hip fractures with delayed diagnosis and treatment had negative outcomes post-operatively, attributed to avoidable immobilization and increased instances of deep vein thrombosis [5].

Where do we go next, especially in the context of evolving and new zoonotic illnesses? First, there is a need to hold extensive discussions on how the medical aspects of these illnesses relate to therapy. For COVID-19, the rehabilitation process itself differs for individuals as the severity of the disease is variable. In the initial phase, if patients are unable to tolerate continuous aerobic exercises, interval training with multiple rest breaks should be carried out. We recommend mobilization with close pulse oximetry monitoring for the hospitalized, regardless of their phase of recovery, to allow for early detection of exercise-induced hypoxemia. The patient’s heart rate should be kept at < 70% of their heart rate reserve during these exercises. Supplementary oxygen may be provided during therapy or at rest for those with exertional dyspnea. With persistent dyspnea, patients might benefit from respiratory muscle training with a threshold positive expiratory pressure device, although specific guidelines for exercise prescription have not been sufficiently investigated. Furthermore, proactive multidisciplinary family meetings to enhance communication with those closest to the patient, as well as establishing their goals of care are crucial in facilitating recovery. It is also imperative to understand indications for referral to rehabilitation services for patients under isolation, and avenues for imparting knowledge and awareness to patients, caregivers, and clinicians even when no hospitalization takes place. Barriers to implementing necessary rehabilitation, such as a shortage of specialists and training gaps for therapists, will need to be addressed as well, especially in the context of new and upcoming zoonotic pandemics, by improving leadership, introducing training workshops, and updating curricula for local preparatory schools. The role of nutrition in preventing and reversing sarcopenia would also need to be quantified to eventually maximize health outcomes for the most vulnerable.

Conclusion

We have highlighted how the geriatric population, especially those with many comorbidities, remains particularly vulnerable when infected and hospitalized for a prolonged course of treatment. Neuroinflammation, sarcopenia, and poor nutrition can contribute to their slow recovery. Rehabilitation remains vital and might be the ‘missing’ link in helping achieve functional and cognitive recovery from COVID-19 and other similar zoonotic illnesses. Future studies should explore the long-term benefits of structured rehabilitation interventions in post-pandemic recovery plans.
